# Neighborhood Revitalization and Cardiovascular Disease Outcomes in Midlife and Older Adults Living in Low-Income Neighborhoods in the Bronx, New York: Protocol for a Natural Experiment and Multimethod Community-Based Study

**DOI:** 10.2196/89056

**Published:** 2026-05-14

**Authors:** Earle C Chambers, David B Hanna, David W Lounsbury, Diana Hernández, Qi Gao, Lihua Li, Ryung S Kim, Sean C Lucan, Yan Li

**Affiliations:** 1 Department of Family and Social Medicine, Albert Einstein College of Medicine, 1300 Morris Park Avenue, Bronx, NY, 10461, United States, 1 718-430-3057; 2 Department of Epidemiology & Population Health, Albert Einstein College of Medicine, Bronx, NY, United States; 3 Columbia University Mailman School of Public Health, Columbia University Irving Medical Center, New York, NY, United States; 4 Department of Population Health Science and Policy, Icahn School of Medicine at Mount Sinai, New York, NY, United States

**Keywords:** neighborhood revitalization, built environment, neighborhood effects, land use rezoning, cardiovascular health, multimethod, community-based mixed methods, systems science

## Abstract

**Background:**

Neighborhood revitalization is a process through which land use rezoning and capital investment can spur new resources, such as access to healthful food and amenities for physical activity. While revitalization efforts may promote cardiovascular health, their benefits may not be distributed equally across sociodemographic groups.

**Objective:**

The objective of the study is to apply a socioecological framework that uses a multimethod approach incorporating quantitative data (longitudinal electronic health records and cross-sectional surveys) and qualitative data (longitudinal “walk-a-long” interviews) to examine the short-term effect of neighborhood land use rezoning and revitalization efforts on cardiovascular disease (CVD), CVD-related health behaviors, and access to and utilization of health care. System science methods, namely microsimulation modeling and system dynamics modeling, will be used to assess the long-term effects of land use rezoning policy and revitalization efforts on cardiovascular health and ways to sustain priority health equity goals in revitalized neighborhoods.

**Methods:**

We leverage a land use rezoning initiative in the Bronx, New York, where a largely commercial area is being rezoned along with capital investments to expand healthful neighborhood resources. Using electronic health records from a single hospital system, we will follow cohorts of midlife and older adults (≥50 y) residing in both the rezoned area and a comparison area. We will assess clinically measured incident CVD and other CVD risk factors to evaluate changes in cardiovascular health over time. In parallel, we will conduct a cross-sectional survey and a purposive sampling of patients for in-person “walk-a-long” qualitative interviews to understand how residents perceive neighborhood access to healthful resources after land use rezoning. To estimate long-term effects, we will use a validated microsimulation model to project CVD outcomes and costs. Finally, we will use system dynamics modeling to integrate quantitative and qualitative findings to inform future revitalization and public health strategies.

**Results:**

Midlife and older adult patients (N=10,813) in the intervention area and the comparison area will be followed for approximately 7 years following land use rezoning and revitalization efforts to compare CVD risk between neighborhoods. The cross-sectional survey (n=300) and qualitative assessment (n=36) will increase understanding of perceptions of access to healthful resources and related health behaviors among residents. Systems science approaches will estimate long-term CVD risk and related costs associated with revitalization efforts. An advisory committee of clinical and community stakeholders will assist in interpreting results and developing dissemination strategies for their constituents. This study was funded from January 2023 until December 2026.

**Conclusions:**

This study uses a socioecological framework to provide a novel, transferable method for evaluating the impact of neighborhood revitalization efforts on cardiovascular health by combining methods to examine short- and long-term effects across individual, neighborhood, and structural (system) levels over time. Findings will inform policies aimed at reducing CVD through equitable urban revitalization.

## Introduction

### Background

Cardiovascular disease (CVD) accounts for 1 in 4 deaths in the United States and costs US $199 billion annually in health care–related expenses in 2018 [1]. Costly CVD-related risk factors, such as hypertension and diabetes, emerge in midlife individuals (aged 50‐64 y), reducing the likelihood of healthy and independent aging into older adulthood [2]. Racial and ethnic disparities in CVD risk factors also widen in midlife [3]. Black and Hispanic populations are at higher risk than White populations for hypertension, obesity, diabetes, and other risk factors for CVD [4]. The burden of CVD is also concentrated among the poor, as CVD is tied to poverty [5]. Related to sociodemographic differences, neighborhood characteristics matter [6]. Higher-poverty neighborhoods with predominantly Black or Hispanic residents are less likely to have access to healthful resources that support behaviors protective against CVD [7-9]. Neighborhoods with less access to healthful food and/or fewer options for physical activity are of particular concern for CVD [9-11].

Large-scale neighborhood revitalization is increasing nationwide, particularly in urban areas [12]. Neighborhood revitalization efforts offer opportunities for better health in under-resourced areas, but perhaps not for all residents. Neighborhood revitalization (also referred to as gentrification, urban renewal, and community development) is a process by which low-income neighborhoods undergo transformation through mechanisms such as land use rezoning, new construction, and renovation. These mechanisms can include large capital investments that spur economic development, upgrade existing physical landscapes, and improve safety. Revitalization efforts can change under-resourced neighborhoods for the “better,” increasing resources to support health [13]. Studies of neighborhood revitalization efforts show potential for lowering the risk of or slowing the onset of CVD [14]. However, study results are mixed, showing both positive and negative effects on health [13,15]. There are many potential reasons for these mixed findings, ranging from study design and rigor to population dynamics that vary by demographic groups [14].

Research shows that older adults can be more dependent on their immediate neighborhood surroundings for food, exercise, and social networks than younger residents [16,17]. However, the needs of older adults are often left out of revitalization efforts [18]. As a result, “aging in place” as the neighborhood changes may come at a cost to health when, for example, the costs of living make accessing these new resources difficult [19,20]. As it relates to older adults, some research suggests an important distinction between “aging in place” and being “stuck in place” as neighborhoods change due to redevelopment [19,20]. “Aging in place” refers to remaining in a community and potentially benefiting from the resources a neighborhood provides. In contrast, “stuck in place” is not being able to leave a neighborhood, for reasons largely related to income, even when the changes are to an individual’s detriment. As neighborhoods attract new resources and often more affluent or younger residents, older adults may experience social isolation [21,22], higher levels of anxiety, and increased depression [19]—all of which are risks for CVD. Research also shows that for Black residents, neighborhood redevelopment may not engender health benefits. Black residents are more likely to report poorer health in changing neighborhoods, whereas other racial and ethnic groups in those same neighborhoods report no difference [23]. Gaps in evidence remain regarding which populations benefit most from these revitalization efforts and under what circumstances the benefits are optimized [13,19]. To address these gaps, there have been calls for natural experiments and longitudinal designs [14]. Furthermore, more qualitative research is needed to better understand how revitalization efforts can be more inclusive of vulnerable older residents so as not to exclude them by neglecting to facilitate access to new healthful resources.

It is evident from the mixed results of prior studies that the evolution in neighborhoods resulting from changes in land use or other neighborhood revitalization policies is complex, with multiple factors likely playing a role. It is also evident that a single method would not sufficiently capture changes in CVD risk over time. To address some of the limitations of prior studies, our study uses a socioecological framework, acknowledging that individual and community factors influence risk for CVD, to inform data collection from multiple methods (quantitative and qualitative) and data integration (systems science) to examine the short-term and long-term effects of land use rezoning policy on CVD among mid-life and older adults.

### Specific Aims and Objectives

The specific aim of this comparative case study is to examine the effect of a neighborhood revitalization effort, starting with a land use rezoning policy, on incident CVD, CVD mortality, quality-adjusted life years (QALYs), and health care costs. The study will follow residents receiving health care from a large hospital system residing in 2 Bronx County, NY neighborhoods: one neighborhood that underwent a revitalization effort (Jerome Avenue) and a comparison neighborhood that is slated to undergo a similar revitalization effort that has not yet occurred (Southern Boulevard). The objective of the study is to use a multimethod approach using quantitative data (longitudinal and cross-sectional surveys), qualitative data (longitudinal qualitative “walk-a-long” interviews), and systems science (microsimulation and system dynamics models (SDM), to understand neighborhood revitalization strategies that best achieve and sustain priority cardiovascular health equity goals and inform clinical social care initiatives. An advisory committee (AC) of relevant stakeholders will be assembled to provide needed expertise on the multiple communities affected by these neighborhood-level policies.

## Methods

### Overview

To address the limitations of prior studies, our study uses a socioecological framework, acknowledging that individual and community factors influence CVD risk, to inform a comprehensive multimethod approach incorporating quantitative data (longitudinal electronic health records [EHR] and cross-sectional surveys) and qualitative data (longitudinal “walk-a-long” interviews) to examine the short-term effect of neighborhood revitalization efforts on CVD, related health behaviors, and access to and utilization of health care. System science methods, namely microsimulation modeling and system dynamics modeling (SDM), will be used to project long-term revitalization effects on cardiovascular health and to explore strategies that sustain priority health equity goals in revitalized neighborhoods. This protocol is described in accordance with the STROBE (Strengthening the Reporting of Observational Studies in Epidemiology) guidelines (Checklist 1) for the EHR and cross-sectional survey components of the project [24].

### Ethical Considerations

This study was approved by the Albert Einstein College of Medicine Institutional Review Board (2022‐14409). Informed consent will be obtained from all recruited participants prior to their involvement in the primary data collection aspects of the study. Financial compensation will be commensurate with the payment schedule described in the *Survey Sample* and *Qualitative Walk-A-Long Sample* sections. Participants may withdraw from the study at any time without penalty or disruption to their health care. To safeguard patient information, data will be deidentified when reported in publications.

### Study Setting and Design

**Figure 1.**
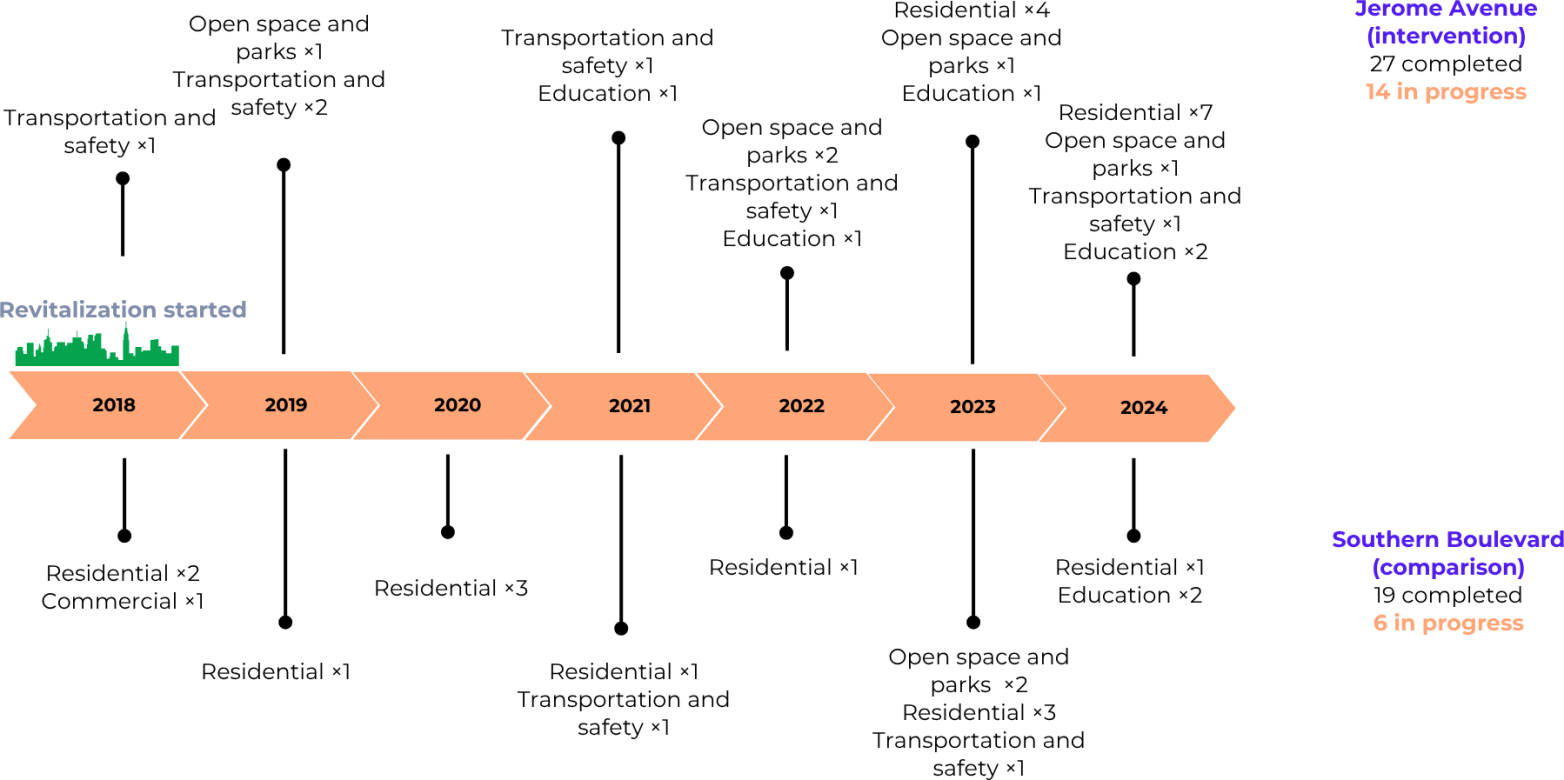
Revitalization and construction projects timeline as of December 2024.

In the Bronx, New York, large-scale neighborhood revitalization efforts provide an opportunity to examine CVD-related outcomes over time. The Bronx has the highest rate of poverty in New York State and is comprised primarily of Black and Hispanic residents. As part of the PLACES (planning for livability, affordability, community, economic opportunity, and sustainability) initiative, New York City has planned extensive revitalization efforts in specific areas of the Bronx, beginning with a land use rezoning [25]. Jerome Avenue is one such area and is the target area for this study. The area was previously largely zoned for commercial, industrial, and auto-related uses. Land use rezoning became effective as of March 22, 2018. According to the NYC Department of City Planning, the revitalization effort will create several important changes, including program development and construction of new affordable housing, new community facilities, and new parkland, and support for small businesses and entrepreneurs in the area (Figure 1).

**Figure 2.**
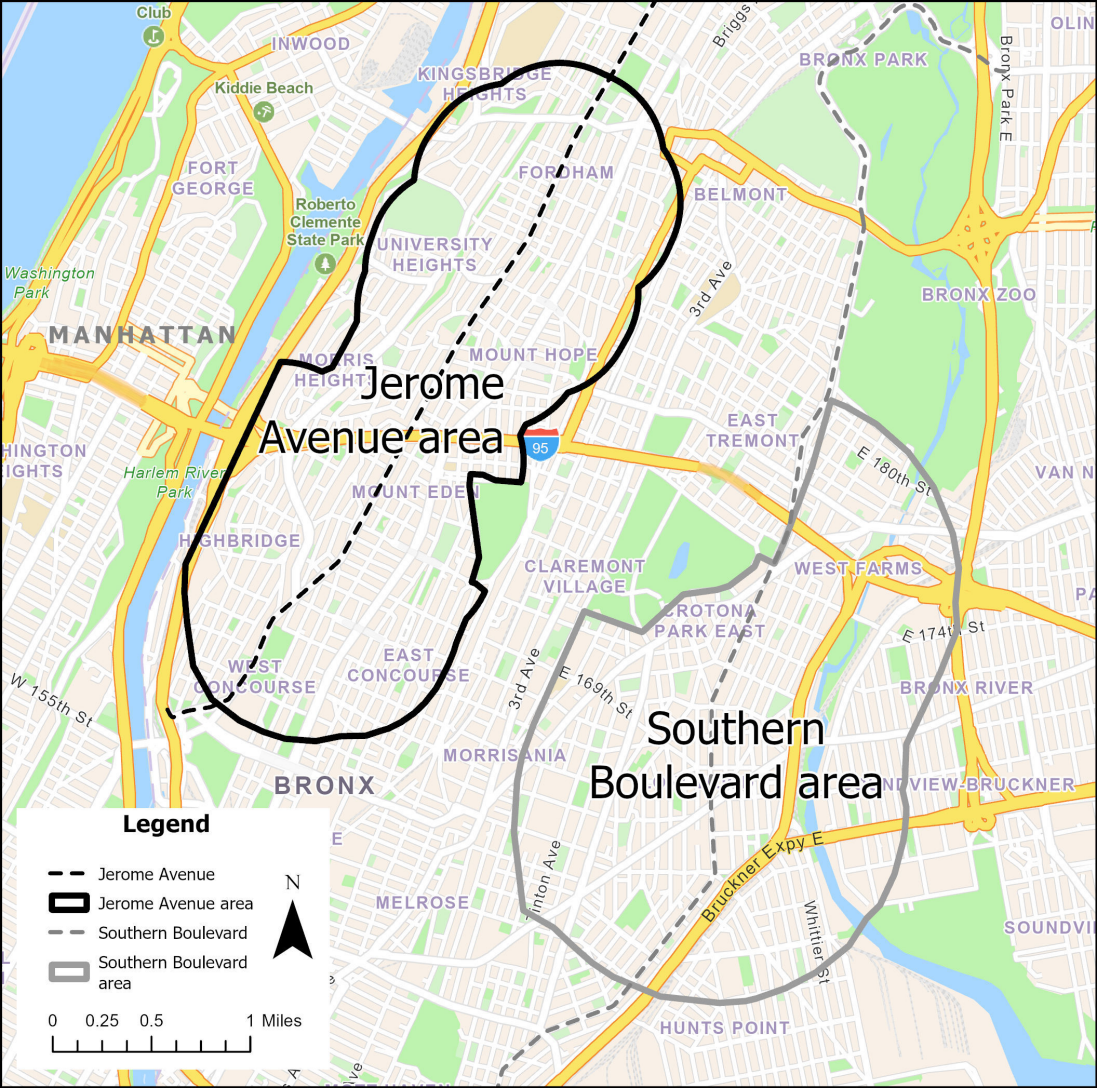
Study area map.

This study follows a cohort of patients residing in the intervention area (Jerome Avenue) and a comparison area (Southern Boulevard) with health measures prerezone and postrezone implementation to investigate how neighborhood revitalization affects CVD and CVD-related risk factors. Patients in the study areas will come from a single health system in the Bronx, New York. Montefiore Health System is a large, integrated health care system that provides primary, secondary, and tertiary care to 860,000 inpatients, with over 5 million outpatient visits annually, serving approximately 32% of Bronx residents. We will follow cohorts of residents in both the intervention and comparison areas from the time of initiation of land use rezoning at the intervention site to the end of the study window, December 31, 2024. The study areas are shown in Figure 2. This study was approved by the institutional review board at the Albert Einstein College of Medicine.

### Study Areas

#### Jerome Avenue (Intervention) Study Area

The NYC Department of City Planning proposed changes to land use that would rezone a 92-block area of approximately 0.24 square miles. On March 22, 2018, the New York City Council approved the land use rezoning of this area of the Bronx. To revitalize the neighborhood, the city committed US $189 million to add and preserve affordable housing, foster economic development, build walkable and recreational spaces, and increase community investment. The project aimed to add 4600 housing units, 1500 of which would be allocated for affordable housing. The land use rezoning plan was scheduled to last 7 years, ending in summer 2025, but due to delays, including the city shutdown due to the COVID pandemic, some projects are still in progress.

#### Southern Boulevard (Comparison) Study Area

The comparison area is a neighborhood located about 1.5 miles to the east of the intervention area that was also considered for land use rezoning, providing a unique opportunity as a comparison area. A formal land use rezoning plan was considered for the comparison area, but approval had not been secured at the time this study was conducted. Some changes in the comparison area’s resources have occurred over time that speak to the fluid and dynamic nature of neighborhoods regardless of concerted revitalization efforts.

The patients residing in these 2 areas have a similar sociodemographic profile with comparable area-level socioeconomic status (Table 1).

**Table 1.** Patient cohort demographics by study area at baseline (2016‐2018).

Demographics	Jerome Avenue study area^a^ (n=7413)	Southern Boulevard study area^a^ (n=3757)	Bronx County^b^
Age (y), mean (SD)	69.8 (9.2)	70.6 (9.7)	44.7 (15.6)
Sex (%)			
Female	68.1	69.3	54.2
Race or ethnicity (%)			
Hispanic	53.2	53.7	54.1
Non-Hispanic Black	31.8	34.3	30
Non-Hispanic White	1.4	1.1	10.3
Other	13.6	10.9	5.5
Insurance status (%)			
Medicaid	27.8	24.2	30.6
Medicare	26.9	29.8	12.1
Commercial	36.4	38.6	46.7
Other	8.9	7.4	10.6
Area poverty (%)^b^^,^^c^	36.9	37.1	27.6

a Data for patients aged 50 years or older from Montefiore Health System.

b From 2012 to 2016, American Community Survey microdata (entire population).

c Area poverty reflects the percent of residents in the study area living below the federal poverty level.

### Sample Eligibility, Recruitment, and Data Collection

#### Patient Sample

**Figure 3.**
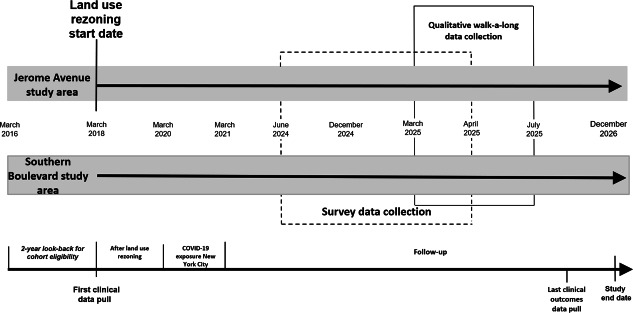
Diagram of data collection during the study period by study area.

This study will use baseline EHR data collected before land use rezoning, as well as 7 years of follow-up EHR data. It will also collect current survey and qualitative data directly from patients during the period following land use rezoning. A description of the survey and qualitative data collection procedures is provided below in the survey sample and qualitative walk-a-long sample sections. The data collection timeline is shown in Figure 3. All EHR data will be deidentified and secured in databases with password protection.

#### Eligibility Criteria

The set of criteria to determine cohort eligibility is shown in Textbox 1. The study sample will include midlife and older adult patients (aged ≥50 y) having at least 1 primary-care outpatient visit between January 1, 2016, and March 22, 2018—approximately 2 years prior to the initiation of the land use rezoning—and who are free of a qualifying CVD diagnosis (exclusion criteria; Table 2). An approximately 2-year qualifying window will ensure a sufficient sample size in each cohort. Primary care outpatient visits will be defined as visits to departments of internal medicine, family medicine, and nonspecialty obstetrics or gynecology, and will include 46 clinical offices at 21 locations. Patients included in the study will also be required to have a residential address documented in the EHR within the 2-year qualifying window prior to the land use rezoning date, located within a one-fourth mile radial buffer around the rezone site (2.6 m^2^) or within a one-fourth mile radial buffer around the proposed comparison site (2.4 mi^2^). The residential address of the closest visit encounter before March 22, 2018, in the 2-year qualifying window was geocoded to confirm that patients live in the study areas.

Textbox 1. **Eligibility criteria**

- Patient must have at least 1 primary care outpatient visit at Montefiore Health System between January 1, 2016, and March 22, 2018 (baseline window).
- Aged 50 years or older and nonpregnant.
- Patient must live within one-fourth mile radial buffer around the Jerome Avenue land use rezoning or within one-fourth mile radial buffer around the Southern Boulevard comparison area.
- Patient must not have a cardiovascular disease diagnosis before March 22, 2018; defined using the *International Statistical Classification of Diseases and Related Health Problems, 10th Revision* (ICD-10) codes for heart failure, heart disease, or stroke.
- Patients that died before March 22, 2018, would be excluded.

**Table 2.** Cardiovascular disease (CVD) diagnosis codes and exclusion criteria for sample eligibility (*International Classification of Diseases, 10th Revision, Clinical Modification* [ICD-10-CM] and *International Classification of Diseases, 9th Revision, Clinical Modification* [ICD-9-CM] codes).

Condition	Exclusion criteria ICD-10-CM	Exclusion criteria ICD-9-CM
Heart failure	I09.81, I11.0, I13.0, I13.2, I50.x	398.91, 402.01, 402.11, 402.91, 404.01, 404.03, 404.11, 404.13, 404.91, 404.93, 428.xx
Ischemic heart disease	I20.x-I25.x	410‐412, 414
Stroke	I60.x-I69.x	430‐438

#### Survey Sample

Of the study sample identified through the EHR for analysis, a subsample of 300 patients (n=150 in the intervention area; n=150 in the comparison area) will be recruited for participation in our study survey. The survey will include 21 domains (Multimedia Appendix 1) measuring demographics, self-reported health measures and morbidity, self-rated health, housing status including residential mobility, energy insecurity, perceptions of the neighborhood environment including walkability and food access, health care access and utilization, social isolation, social networks, health behaviors including sleep duration and quality, tobacco use or smoking, daily alcohol use, sedentary behaviors, physical activity, and food insecurity. The sampling strategy will include random stratified sampling in each study area from strata of age (50-64 and ≥65 y), sex (male and female), and area deprivation index (ADI; low: 0‐20 and high: ≥20). Study coordinators will contact eligible patients first by email and then subsequently by phone following the random order of patients in each stratum until the required number of patients is reached. Study coordinators will follow the outreach protocol to confirm eligibility and complete informed consent in English or Spanish. Participants will be offered US $60 for completing a 1.5-hour survey questionnaire. Participants will sign informed consent before recruitment and data collection.

#### Qualitative Walk-a-Long Sample

Patients enrolled in the survey sample will be recruited for inclusion in the qualitative walk-along portion of the study. The qualitative sample will include a purposive sampling of patients stratified by sex, age, and ADI. A total of 36 participants will provide informed consent (n=24, patients from the intervention area; n=12, from the comparison area). Repeated interviews, field observations, and images, along with oversampling participants from the intervention area, will allow for a nuanced exploration of the residents’ experiences regarding neighborhood changes and how those changes might be connected with health behaviors relevant to CVD risk. The qualitative interviews for the study will take place in residents’ neighborhoods, while walking with trained staff and at residents’ homes [26,27]. The researcher will seek to describe the very essence of a shared experience—in this case that of neighborhood revitalization. The researcher will use an interview guide with designated themes to be covered during each session to ensure that key topics are discussed and revisited during follow-up. The interview guide will contain 7 domains (Multimedia Appendix 1) to allow for a comprehensive assessment of neighborhood perceptions, household and housing characteristics, changes in routine and physical activity, the food landscape, and financial stability from the point of view of midlife and older adults. The baseline interview will be conducted by walking in the neighborhood, followed by one follow-up at residents’ homes and a second follow-up in another neighborhood walk-along. The proposed subsample of 36 patients will complete interviews that last about 60 minutes. Each interview will take place 6 months apart to measure perceptions at different points in time. The walking interview will be conducted in the spring or fall to accommodate outdoor temperatures and will be done in person in English or Spanish, depending on patient preference. The patients will be video- and audio-recorded while walking in their corresponding neighborhoods and at home for a total compensation of US $300. The recordings will then be transcribed for analysis using NVivo software [28].

### Data Analysis Plan

#### Overview

Our analysis plan follows from the aim of the study, which examines short-term CVD risk associated with neighborhood revitalization using clinical data; the use and access to healthful neighborhood resources after land use rezoning using data from a cross-sectional survey and qualitative assessment; and the long-term effects of revitalization on CVD using system science approaches (ie, microsimulation analysis and SDM).

#### Short-Term Effects of Neighborhood Revitalization on CVD Using EHR Data

To examine the CVD risk in 2 study areas, we will define the primary end point as the time from the start of land use rezoning (March 22, 2018) to the date of the first composite CVD incidence. For each patient, the end of follow-up will be defined as the earliest among (1) the date of the first composite CVD incidence (ie, the outcome event), (2) the date the patient died or moved out of the study area, or (3) December 31, 2024. We will summarize the risks using the Kaplan-Meier estimates of the incidence-free probability in each area. To compare the difference in CVD risks between the 2 study areas, we will perform a 2-sided Cox regression with a significance *α* level of .05, using the binary indicator of the residing area on March 22, 2018, as the predictor of interest. Models will be adjusted for potential confounders such as age, sex, diabetes history at baseline, smoking status at baseline, insurance type, race, and neighborhood ADI score. As a secondary analysis, an “intention-to-treat”-like analysis will be done by defining the end of follow-up as the earlier date between (1) the date of the first composite CVD incidence or (2) December 31, 2024, ignoring whether the patients moved out of the study area during the follow-up period. To check model assumptions, we will perform 3 types of diagnostics for Cox regression models: testing the proportional hazards assumption (using graphical methods and formal tests based on scaled Schoenfeld residuals), examining influential or outlying observations (using graphical examination of deviance residuals), and detecting nonlinear relationships between the log hazards and the covariates (using graphical examination of Martingale residuals) [29]. If the proportional hazards assumptions are violated, we will build a time-dependent hazard ratio model using time-varying covariates, specifically the interaction term between time segments and the indicator of the study area. The sample size consists of 7413 and 3757 patients who resided on Jerome Avenue and Southern Boulevard, respectively, on March 23, 2018. [30]

### Outcome Measures

#### Primary Cardiovascular Disease Outcome

The cardiovascular disease outcomes occurring after March 22, 2018, will be defined using *International Classification of Diseases, 10th Revision, Clinical Modification* (ICD-10-CM) diagnosis codes (Table 2) to identify incident congestive heart failure, ischemic heart disease, and stroke. A composite CVD measure, including the occurrence of any CVD event mentioned above, will be used in analyses.

#### Secondary Outcomes

Allostatic load will be measured using the Index for Cardiometabolic Health (ICMH), consistent with a strategy validated by Nobel et al [31] using estimated glomerular filtration rate, serum albumin, BMI, mean arterial pressure, hemoglobin A_1c_ (HbA_1c_), total cholesterol, high-density lipoprotein cholesterol, low-density lipoprotein cholesterol, triglycerides, and urine albumin/creatinine ratio. The ICMH is a z-score–based approach to measuring allostatic load using EHR data that standardizes each component measure based on the sample mean on a scale of 0 to 100, with a mean of 50 (SD 12.5). The mean of the component measures is the overall ICMH score. Missed clinical visits will be defined as no-shows at clinical visits including same-day cancellations, but excluding visits canceled due to changes in provider schedules. This outcome definition has been used previously in analyses using data from our hospital system [32].

#### Covariates

EHR data includes age at baseline, gender, race, ethnicity, and type of insurance. All covariates are collected during patient clinical visits. Height, weight, systolic blood pressure, and diastolic blood pressure measured at clinic visits identified within the EHR will be used in analyses. Height and weight will be used to calculate BMI using weight in kilograms divided by the square of height in meters. Recorded sex, race, and ethnicity are based on how a patient self-identifies. Insurance type is based on billing information. Because income is not systematically recorded in the EHR, we will use insurance payers (ie, Medicaid, Medicare, and commercial) as a proxy measure, acknowledging its limitations as a measure of socioeconomic status. Census tract-based ADI, as developed from the American Community Survey by the Neighborhood Atlas (University of Wisconsin-Madison) [33], will be derived after obtaining census tract information by geocoding residential addresses. ADI is a commonly used measure of neighborhood deprivation using US Census data at the census tract level and has been shown to correlate with CVD and related risk factors such as BMI and blood pressure [34].

#### Power Calculations

From the Hispanic Community Health Study/Study of Latinos [35], the incidence rate of CVD in Bronx participants between their baseline interview (2008‐2011) and visit 2 (2014‐2017) for those aged more than 50 years is 42 per 1000 person-years. Assuming a hazard rate of 0.042 per year in the comparison area and a conservative 20% attrition rate, the proposed sample size (n=7413 from Jerome Avenue; n=3757 from Southern Boulevard) for a 2-sided test in the Cox proportional hazards model has more than 80% power to detect a CVD hazard ratio of 0.886 between the 2 study areas at a significance level of .05 with approximately 7 years of follow-up.

#### Missing EHR Data

All data analyses will be preceded by extensive data checking and verification to identify and resolve the reasons for missing values, inconsistencies, and out-of-range values. Models proposed for analysis can address incomplete data but do require, at least, that missingness be at random. We will carefully examine whether it is missing completely at random, missing at random, or missing not at random, and modeling will consider the use of multiple imputation techniques for covariates to reduce potential biases. When the missing at random assumption is violated, we will include in the imputation model variables that assess overall health status (eg, Elixhauser Comorbidity Index [36]) and health care use. We acknowledge that the probability of missingness is likely associated with disease severity and the number of clinical encounters, since unhealthier patients are more likely to have comorbidities, and each interaction with the health care system provides an opportunity for documentation [37].

### Utilization and Access to Neighborhood Healthful Resources Due to Neighborhood Revitalization Using Cross-Sectional Survey and Qualitative Data

#### Survey Data Analysis Approach

Data collected from the survey of 300 participants will be used to compare cross-sectional differences in health behaviors such as diet, physical activity, smoking, and alcohol consumption between the study areas approximately 7 years after the land use rezoning. We will then use the chi-square test to compare incidence proportions of CVD outcomes for participants in each area and a Student 2-tailed *t* test or Mann-Whitney *U* test to compare continuous variables. Subsequently, we will perform regression analysis to adjust for covariates. Based on previous analyses of the Bronx population, the expected rate of leisure-time physical activity in the comparison area is estimated to be 64.7%. We will perform a 2-sided Wald test with logistic regression, using leisure-time physical activity as the binary response variable, the binary indicator of study area as the predictor of interest, and the potential confounders as covariates. The proposed sample size (n=150 from Jerome Avenue; n=150 from Southern Boulevard) yields a minimum detectable leisure-time physical activity rate in the intervention area of 79.2% [38] at 80% power and a 5% level of significance.

#### Qualitative Data Analysis Approach

Interview recordings from qualitative data collection will be transcribed verbatim, standardized to the English language, and analyzed as textual and visual data along with field notes using the qualitative software package NVivo (version 15) [28]. The analysis will combine an inductive approach to identify emergent themes and patterns that might arise from the data as well as a deductive approach to support an integrated interpretation of quantitative findings. Following this framework, at least two members of the research team will conduct a 2-phase inductive analytical process informed by the phenomenological approach [39]. The initial analysis will involve “open coding” in which all transcripts and visual documents will be coded to perform a descriptive assessment of the data, following the 7 domains in the interview guide, as well as emergent issues shared organically by participants and through probing (Multimedia Appendix 1). The second phase will entail “axial coding” to examine relationships among codes and identify themes based on shared and complementary characteristics of codes. For inductive analyses, we will describe key themes that are frequently repeated and conceptually meaningful to understand redevelopment and health with a focus on lived experience and the essential elements of this phenomenon. For deductive analyses, codes will be thematically grouped, and patterns in the data will be assessed to reflect causal relationships, contextual factors, intervening conditions, strategies, and consequences. Particular attention will be given to possible mechanisms through which neighborhood conditions (enablers or barriers), such as perceived safety, environmental stressors, and access to health-promoting resources, may influence CVD-related outcomes, including gradual changes in physical activity, the food landscape, healthful resources, and engagement with the community. We will draft analytic memos to document coding decisions and logic related to the interpretation of data. Findings will be reported using representative quotes and thick descriptions of participant accounts and neighborhood observations based on repeated interviews, observations, and images. NVivo, a qualitative data analysis software package, will be used to manage interview transcripts, field notes, and images, and facilitate coding and thematic analysis. To strengthen the interpretation of findings, triangulation methods will be used to integrate qualitative insights with quantitative results. We will present preliminary findings to the study’s community advisory board and willing study participants as a form of member checking, ensuring that interpretations are validated by participants and other community members.

### Long-Term Effect of Neighborhood Revitalization on CVD Using Microsimulation Analysis

In addition to the short-term effects of the revitalization effort on CVD outcomes and related health behaviors, this study will use microsimulation methods to predict the long-term effects of neighborhood revitalization on CVD events, CVD mortality, QALYs, and health care costs. Computer simulation models have been used to inform chronic disease prevention [40,41]. Some of these simulation models have been used by local health departments to facilitate and inform decision-making—for example, to predict population health trajectories, engage community members and other stakeholders with data visualization and animation around health outcomes, evaluate intervention programs, and establish evidence for future implementation of programs to improve population health [40,42]. In addition to projecting the effects of policies and interventions, we use rigorous validation procedures to ensure the results are robust. To predict the long-term effects of revitalization efforts on reducing CVD-related outcomes and associated health care costs, we will expand the NYC version of our CVD model, which projects incidence, prevalence, mortality, and costs associated with heart failure, stroke, and coronary heart disease in adults that are difficult to measure due to the typically long latency period of CVD.

We will use results from our analysis of EHR data as the inputs for the simulation to create a simulated population that is representative of the midlife and older adult residents living in the study areas. The model can then simulate a “revitalization” scenario and a “nonrevitalization” scenario. Under each of the 2 scenarios, we will project CVD outcomes (ie, number of heart failure, coronary heart disease, stroke events, and deaths due to CVD), QALYs, and associated health care costs over 10 years, 15 years, and 20 years, and the lifetime of the simulated population. This will allow us to examine how many cases of CVD the neighborhood revitalization would have prevented and how much health care costs the intervention would have saved in the long term. We will divide the difference in costs by the difference in QALYs to calculate the incremental cost-effectiveness ratio of the revitalization intervention compared to the “nonrevitalization” scenario. To determine whether the cost of neighborhood revitalization is worth the health benefits gained, we will use cost-effectiveness thresholds recommended by the American College of Cardiology and the American Heart Association [43]. These recommendations specify an intervention with an incremental cost-effectiveness ratio of less than US $50,000 per QALY gained as the threshold for a high-value intervention.

For model validation, we will compare the baseline characteristics between the simulated population and the cohort using the collected EHR data. Moreover, we will conduct predictive validation by comparing simulation results with collected data on CVD incidence. Specifically, we will compare the 1-year and 5-year incidence and prevalence of CVD that our model projected with the observed values from EHR data to calibrate and validate our model until our CVD model demonstrates that it can produce outcome predictions within a reasonable error margin compared to real-world data. Additionally, we will compare our study area simulation results with other published studies, as well as external data from the CDC WONDER database. The simulation is run 1000 times to generate more stable and less biased estimates.

We will calculate the effect of neighborhood revitalization on CVD outcomes among 3 racial and ethnic groups: non-Hispanic Black, non-Hispanic White, and Hispanic. The results will show whether neighborhood revitalization has reduced or increased long-term health disparities among these racial and ethnic groups. A similar analysis will be conducted by sex (eg, male vs female).

### Plan for Integration of Qualitative and Quantitative Data to Achieve and Sustain Priority Health Equity Goals Regarding Access to and Use of Neighborhood Healthful Resources Using SDM

SDM will be used to examine short-term as well as long-term impacts on the health and well-being of people living in revitalized neighborhoods, with the aim of informing strategies that sustain priority health equity goals in these neighborhoods. SDM will serve to compare and contrast results and key insights from our qualitative and quantitative analyses, which will be derived from multiple empirical data sources (survey results, clinical data results, observational findings from qualitative data, and microsimulation modeling projections of long-term health outcomes).

Best practices in SDM methods apply group model building (GMB) that engages key stakeholders in a multistep, iterative procedure involving problem identification, system conceptualization, model formulation, model simulation, and model evaluation [44-47]. In this study, key stakeholders will include all co-investigators as well as members of our community AC. Our community AC will include persons with expertise in NYC government, community-based organizations, and individuals who live and work in the neighborhoods targeted in the study. Selected GMB exercises [48] will be used to foster the design, development, and application of SDM that will, via model-generated sensitivity analyses and comparative scenario assessment, inform questions about how health and well-being change among people 50 years and older who live in revitalized neighborhoods over time.

We expect that SDM will provide a novel way to synthesize findings generated from each of the methods that comprise this study. For example, our SDM will embroider on (1) quantitative outcomes (effects) of our proposed midlife and older adult cohort comparison in the study areas; (2) qualitative themes derived from purposive sampling of residents engaged in our proposed “walk-a-long” and key informant interviews; and (3) long-term trend analyses informed by microsimulation modeling for assessment of CVD incidence, prevalence, and associated health system costs. Resultant SD models will be used to identify and test, via simulation analyses, study-supported insights about effective future public health strategies and ways to cultivate and sustain priority health equity goals in these neighborhoods.

**Figure 4.**
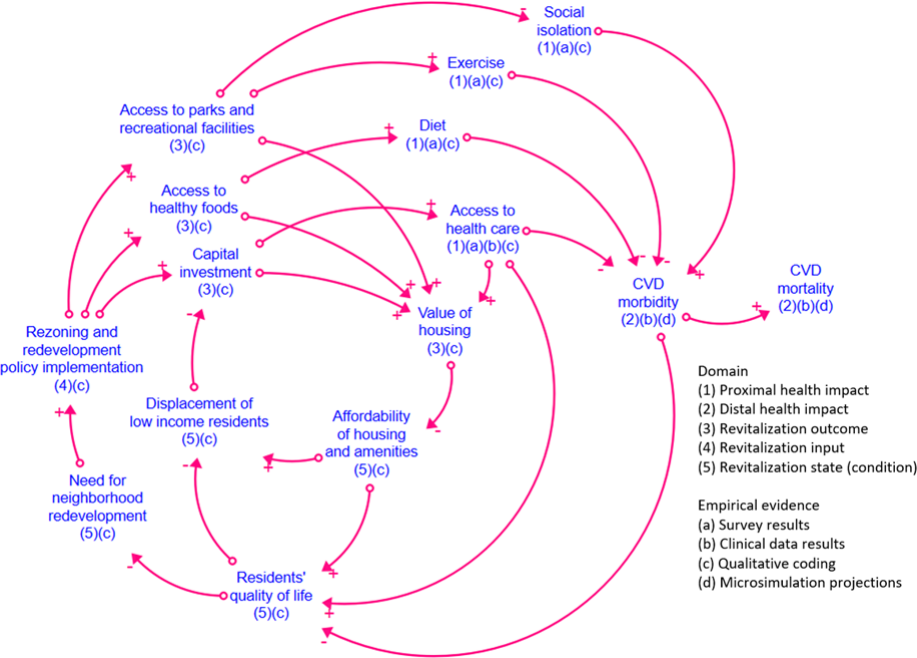
Causal loop diagram of hypothesized key drivers of cardiovascular outcomes among midlife and older adults in our intervention neighborhood. Every causal link has a polarity, either positive (+) or negative (−). A positive (+) link indicates that an incremental increase (or decrease) in the antecedent variable causes the target variable to also increase (or decrease), while a negative (−) link indicates the opposite relationship between two variables. Defined feedback loops represent a theory of change, which can be translated into a testable computational system dynamics modeling (SDM). CVD: cardiovascular disease.

Our framing research question will be to use SDM to explain: “How do older adults seek to live healthful lives in their neighborhoods?” GMB exercises with study co-investigators and collaborating community partners will begin by critiquing a preliminary qualitative model, or causal loop diagram (CLD; Figure 4). Systems thinking exercises will be used to refine this CLD. For example, results from qualitative themes and key informant interviews can be used to support SDM that demonstrates how revitalization policies may impact residents’ decision-making regarding eating, exercise, access to resources, and cardiovascular morbidity and mortality over time. Our CLD includes preliminary feedback loops that define our initial hypothesis about key drivers of cardiovascular outcomes among midlife and older adults in our intervention neighborhood. Defined feedback loops represent a theory of change, which can be translated into a testable computational SDM. Computational SDM produces a set of differential and algebraic equations, rendered as a stock-and-flow diagram. The envisioned computational SDM will allow for simulation analyses that will support scenario-based comparative effectiveness assessments and sensitivity analyses of land use rezoning and revitalization policy outcomes on social isolation, routine exercise, and diet, and, in turn, on CVD morbidity and mortality rates in the study neighborhoods.

Working collaboratively with study co-investigators and applying best practices in SDM methods, we will review and apply findings from other data streams in this study. We intend to use a convergent approach to support our SDM, where qualitative and quantitative data are collected simultaneously, analyzed separately, and then integrated to inform the interpretation of findings and triangulation of results. This study applies different methods, including qualitative research methods, the results of which are used to embroider upon our initial CLD. The resultant CLD will be used to show the contributions of these methods, producing a synthesized hypothesis of the dynamics driving health impacts in study populations exposed to land use rezoning and neighborhood revitalization. The multiple pathways that influence the development of cardiovascular outcomes can be captured by this simulation, reflecting the changes in CVD morbidity and mortality over time due to neighborhood revitalization efforts. The simulation results will be evaluated by gathering feedback from the study team with the goal of describing promising strategies, recommendations, and emerging priorities for future neighborhood revitalization planning. All SDM will be designed, evaluated, and disseminated using the Stella Architect software package (isee systems, inc) [49].

## Results

This study was funded from January 2023 until December 2026. The baseline characteristics of eligible patients in each of the study areas, identified through the EHR for analysis and further recruitment for survey and qualitative assessment, are presented in Table 1. A sample of 10,813 patients aged 50 years or older, identified from the EHR and residing in the study areas, will be followed for approximately 7 years following land use rezoning and revitalization efforts to compare CVD risk between neighborhoods. Of these patients, 300 (n=150 from Jerome Avenue; n=150 from Southern Boulevard) will be recruited for survey measurement, and 36 for qualitative walk-a-long interviews. The patients in the study areas have comparable sociodemographic characteristics. Approximately 95% of the residential addresses were successfully geocoded within New York City, and 90% of patients in the 2 study areas who had multiple addresses documented over the study period remained in the same study area. The dissemination plan includes manuscripts for publication in peer-reviewed health journals and communication engagement strategies for different stakeholders, including community partners. An advisory committee of clinical and community stakeholders will assist in interpreting results and developing dissemination strategies for their constituents.

## Discussion

### Expected Findings

This study demonstrates a transferable method for quantifying the impact of neighborhood revitalization efforts in an area actively undergoing this process, using both clinical and community data. This study will capitalize on a unique natural experiment, use the EHR system to follow patients over time, incorporate data from multiple sources, and use a longitudinal qualitative assessment of neighborhood perceptions among midlife and older adults experiencing a neighborhood transition. The study will also use microsimulation modeling to project long-term CVD-related effects. The SDM will be used to simulate various scenarios of change following land use rezoning, which can inform the rollout of neighborhood revitalization efforts in similar geographic areas.

### Limitations and Challenges

Natural experiments in the context of neighborhood land use rezoning, being quasi-experimental by design, have the limitation of not being able to fully control for other exposures that cohorts may be exposed to during the study period, which could influence changes in health outcomes (ie, residual confounding). There is also a possibility of study participants moving out of the study areas and being lost to follow-up. Since we are following the incidence of CVD using EHR, discontinuing care within the Montefiore Health System or getting care simultaneously at another health system would pose a barrier to collecting follow-up data, making estimates vulnerable to attrition bias. We attempt to understand the potential for bias by confirming results with microsimulation models. The microsimulation model simulates individual-level life trajectories of risk factors informed by the analysis using EHR data, allowing for time-varying covariates and dynamic changes in risk profiles over time. Through rigorous calibration and validation, the model minimizes discrepancies in CVD event incidence and mortality rates between the simulated data and the observed study data (internal data), as well as external data from the CDC WONDER database, thereby reducing potential bias.

Using a clinical sample has the benefit of characterizing smaller geographic areas, tracking participants, objectively measuring clinical outcomes, and collecting repeated measures over time, which are difficult and costly to accomplish through population-based study designs. We believe that the benefits of using clinical data from a large hospital system (supplemented with targeted survey data collection) and following a comparison cohort of patients from a similar neighborhood not undergoing a land use rezoning and revitalization effort far outweigh the limitations. By using survey data and EHR data, we can characterize the potential bias and adjust estimates to gain a better perspective on risk. Our decision regarding study design was made to improve internal validity perhaps at the expense of external validity; thus, these results may not be broadly generalizable to other cities and areas but are rather meant to characterize the impact of local policy on local residents. Our approach leverages data from a health system that represents 32% of the population in Bronx County, which can serve as a model for other large hospital systems to identify high-risk patients using neighborhood as the context for determining risk.

The effects of land use rezoning and revitalization efforts on CVD are complex, with many potential pathways (eg, health behaviors, safety, housing security, and psychological stress). We are using a cross-sectional survey design and a qualitative approach to illuminate some of the key pathways identified by patients. Our microsimulation models can capture the overall effect of multiple pathways on CVD, as any potential improvement in diet and physical activity, for example, due to neighborhood revitalization, will be reflected by changes in CVD risk factors over time. However, we acknowledge that there are limitations to collecting health behavior data in the way proposed and that we are not measuring all possible pathways in this study. In the future, data can be added to our study to better elucidate pathways. For example, new methods are being developed using Google Street View to objectively measure physical characteristics of neighborhoods [50]. More recent work using Google Street View has shown potential to measure change in neighborhood features over time [51].

### Conclusions

The strengths of this study are many. City planning agencies work with city health departments to produce health impact assessments for revitalization projects but are often limited to a small set of health measures and rarely include long-term health outcomes. By focusing on neighborhood land use rezoning and subsequent revitalization efforts, the results from this study can inform both local policies that require data to inform land use rezoning and revitalization efforts, as well as support public health agencies and health systems that require data to develop mitigation strategies to prevent poor health outcomes that may be associated with these efforts. Upcoming planned analyses will further characterize the cardiovascular-related benefits and/or risks associated with land use rezoning and revitalization efforts in the study communities, as well as patients who relocate or are displaced from the study areas after land use rezoning, providing needed perspectives on midlife and older adult residents.

## Supplementary material

10.2196/89056 Multimedia Appendix 1 Cross-sectional survey and qualitative assessment domains and measures.

10.2196/89056 Checklist 1 STROBE checklist.

## Abbreviations

AC: advisory committee
ADI: area deprivation index
CLD: causal loop diagram
CVD: cardiovascular disease
EHR: electronic health record
GMB: group model building
HbA_1c_: hemoglobin A_1c_
ICD-10-CM: *International Classification of Diseases, 10th Revision, Clinical Modification*
ICMH: Index for Cardiometabolic Health
PLACES: planning for livability, affordability, community, economic opportunity, and sustainability
QALYs: quality-adjusted life years
SDM: system dynamics modeling
STROBE: Strengthening the Reporting of Observational Studies in Epidemiology
