# Supplementary material for: Neighborhood Revitalization and Cardiovascular Disease Outcomes in Midlife and Older Adults Living in Low-Income Neighborhoods in the Bronx, New York: Protocol for a Natural Experiment and Multimethod Community-Based Study
Source: JMIR Res Protoc. 2026 May 14;15:e89056. doi: 10.2196/89056 (PMC13175449; doi:10.2196/89056)
Supplement: Multimedia Appendix 1 [file resprot-v15-e89056-s001.docx]

| **I. Cross-sectional Survey Domains and Measures** | |
| --- | --- |
| **Domains** | **Measures** |
| Demographics | -- |
| Height and weight | National Health and Nutrition Examination Survey |
| Morbidity | Hispanic Community Health Study/Study of Latinos |
| Self-rated health | Short Form-36 Health Survey |
| Housing Security | Affordable HOME Study |
| Residential Mobility | Hispanic Community Health Study/Study of Latinos |
| Neighborhood environment walkability | Neighborhood Environment Walkability Scale |
| Health care utilization | National Health Interview Survey |
| Social isolation | UCLA Loneliness Scale |
| Social network | Berkman-Syme Social Network Index |
| Mental health outcomes | Generalized Anxiety Disorder 7-item scale; Patient Health Questionnaire –8 |
| Sleep duration and quality | Pittsburgh Sleep Quality Index |
| Tobacco use/smoking | Population Assessment of Tobacco and Health |
| Daily alcohol use | New York City Community Health Survey |
| Sedentary behavior | PhenX Sitting‑Sedentary Behavior Adult |
| Physical activity | Global Physical Activity Questionnaire |
| Food insecurity | U.S. Household Food Security Survey, USDA |
| Food access | Food Access and Affordability |
| Energy insecurity | Hernandez Household Energy Insecurity Scale |
| Extreme heat adaptation behaviors | Erens et al. BMC Public Health. 21. 2021.  Milando et al. BMC Public Health. 22(1). 2022. |
| Heat-related illness | Milando et al. BMC Public Health. 22(1). 2022. |

**Appendix 1: Cross-sectional survey and qualitative assessment domains and measures**

**Appendix 2.** Survey assessment domains

| **II. Qualitative Assessment Domains** |
| --- |
| **Domains** |
| Home and family |
| Neighborhood perceptions |
| Daily routines |
| Physical activity |
| Recreational/ social activities |
| Health and food |
| Household income and expenses |
